# Esophageal Sarcoidosis: A Review of Cases and an Update

**DOI:** 10.1155/2013/836203

**Published:** 2013-03-05

**Authors:** Albin Abraham, Rabab Hajar, Ravi Virdi, Jaspreet Singh, Paul Mustacchia

**Affiliations:** ^1^Department of Internal Medicine, Nassau University Medical Center, East Meadow, NY 11554, USA; ^2^Department of Gastroenterology, Nassau University Medical Center, East Meadow, NY 11554, USA

## Abstract

Sarcoidosis is a chronic disorder that can virtually affect any organ system in the body. Histologically, it is characterized by the presence of T lymphocytes, mononuclear phagocytes, and noncaseating granulomas. Most commonly affected are the intrathoracic structures, with 90% of the reported cases involving the lungs. Esophageal involvement in sarcoidosis is extremely rare. Dysphagia is the most common presentation in these patients and can be attributed to various mechanisms such as direct esophageal wall infiltration, extrinsic compression, cranial neuropathy, and brainstem involvement. A thorough online literature review revealed only 23 reported cases of esophageal involvement in sarcoidosis. This paper reviews these reported cases in detail along with newer diagnostic and treatment options, including direction of future therapy.

## 1. Introduction

Sarcoidosis is an inflammatory, granulomatous, multisystem disorder of unclear etiology [1]. The lungs are predominantly involved, but it can entail involvement of any other organ or organ systems such as the skin, lymphatics, heart, musculoskeletal, neurological, and gastrointestinal system [2, 3]. The first description of sarcoidosis was published by Jonathan Hutchinson in 1877, where he described a patient with multiple raised purple lesions on his skin [4]. However, it was Caesar Boeck in 1899 who coined the term “sarkoid,” due to the histological appearance of the skin lesion that he thought was similar to sarcoma [5].

Sarcoidosis is a global disease with variable incidences, presentation, and prognosis. It has the highest incidence in the United States and Sweden [4]. In the United States, it is more common in African Americans with an age adjusted annual incidence rate of 35.5 in 100,000, whereas in Caucasians it is 10.9 in 100,000. The lifetime risk of developing sarcoidosis is 2.4% in African Americans compared to 0.85% in whites [6]. Sarcoidosis also has a preponderance to occur more frequently in women as compared to men. Though it can affect any age group, sarcoidosis tends to affect individuals aged 40 years or younger. However, in Japanese and Scandinavian women, the disease process appears to be biphasic with the prevalence most common in the third and seventh decades of life [7]. Clinical presentation also varies among individuals in different parts of the world. Lofgren's syndrome, which is an acute form of sarcoidosis, with patients manifesting with a triad of bilateral hilar lymphadenopathy, arthritis and erythema nodosum, is more common among those hailing from Southern Europe. African Americans are more likely to present with involvement of the liver, eye, bone marrow, and extrathoracic lymph nodes than with erythema nodosum. Japanese patients have higher rates of cardiac and ophthalmologic involvement while patients from Puerto Rico have higher rates of involvement of the skin [8]. Sarcoidosis also seems to carry a favorable prognosis when it occurs in children as suggested by a recent study which showed complete resolution of the disease in 75% of affected Danish children [9]. 

Gastrointestinal tract involvement in sarcoidosis is very rare. Elaborate postmortem studies of patients with sarcoidosis, published between 1949 and 1963, did not find any evidence of GI tract involvement [10–13]. However, studies published later showed the presence of subclinical gastrointestinal involvement of sarcoidosis in 5–10% of patients [14, 15]. The prevalence rates of clinically significant and identifiable gastrointestinal sarcoidosis have been reported to be around 0.1 to 0.9% of patients with the disease [16, 17]. In Japanese patients with sarcoidosis, the prevalence of gastrointestinal sarcoidosis was 1.6% according to a recently published epidemiologic study [7]. Among cases of gastrointestinal tract sarcoidosis, the stomach is the most frequently involved organ, with more than sixty published cases in the literature [18]. These patients frequently present with epigastric pain, hematemesis, malena, nausea, vomiting, and early satiety [19]. 

Esophageal involvement is an extremely rare occurrence in sarcoidosis. A review of the literature revealed only 23 published cases of esophageal involvement in sarcoidosis to date [19–42]. The first case of esophageal sarcoidosis was reported by Kerley in 1948 [19]. The patient presented with symptoms of difficulty swallowing and an esophagram showed moderate stenosis of the distal esophagus. Biopsy of the involved esophageal mucosa showed the presence of a granuloma. The aim of this paper is to review and summarize published cases of esophageal sarcoidosis, its clinical features, diagnosis, and treatment options, including future directions in its management. 

## 2. Pathogenesis

The exact causes of sarcoidosis remain unknown. However, various infectious and environmental antigens have been suggested as triggers, causing sarcoid to develop in a genetically susceptible individual. The immune response in sarcoid is similar to those present in other granulomatous diseases caused by a known antigen, such as tuberculosis and berylliosis. There have been various reports of community outbreaks and clustering of cases in time and space which has led to speculation and suggestions of a possible environmental antigen or even a person to person transmission of sarcoid. There have been reports of increased disease incidence and clustering in certain occupations such as nurses and firefighters [4, 43]. Occupational exposure to pesticides and microbial bioaerosols has also been listed as having a possible association with the development of sarcoidosis [44]. Other proposed causes and associations include radiation exposure, automobile manufacturing, and inhalants such as talc, zirconium, aluminum, pollen, and mold [45]. The apparent transmissibility that has often been observed has lead to postulation that an infectious etiology also contributes to the development of the disease. The two most commonly implicated microorganisms are mycobacterium and propionibacteria. Various studies have shown that sarcoid granulomas contain in it, the presence of mycobacterial nucleic acids and proteins such as *Mycobacterium tuberculosis* katG and *M. tuberculosis topoisomerase* [46]. Similarly *P. acnes* have been frequently detected in patients with sarcoidosis [47]. This was highlighted by a multicenter study which showed the presence of propionibacterial DNA in 98.15% of sarcoidosis lymph node samples [48]. Other infectious agents often linked with sarcoidosis include the Epstein Barr virus, *Cytomegalovirus*, *Mycoplasma*, Herpes virus and Borrelia Burgdoferi [45, 49]. 

The increased occurrence of sarcoidosis among families and an increased incidence specifically among monozygotic twins had led to initial suggestions that genetic susceptibility plays an important role in sarcoidosis. Compared to Caucasians families where only 5% had more than one affected individual, the rates in African American families were as high as 19% [50]. Studies have shown that the overall familial relative risk of having sarcoidosis is 4.7% [44]. As with many other chronic inflammatory diseases, sarcoidosis has also been associated with genes in the major histocompatibility complex (MHC). The HLA-DRB1*1101 allele and the transporter of antigenic-peptide- (TAP-) 2 alleles have been associated with sarcoidosis. Additionally the HLA-DQB1*0201 allele has been associated with a good outcome whereas the HLA-DQB1*0602 has been linked with a poor prognosis. Other genes that have been linked with the development of sarcoidosis include the toll-like receptor 4 gene, transforming growth factor beta (TGF-*β*), interleukin- (IL-) 1*α* and C-C chemokine receptors. The increased levels of angiotensin converting enzyme (ACE) in some patients with sarcoidosis has led to interest and enthusiasm in evaluation of ACE gene polymorphisms. However, most reviews have found no association between these polymorphisms with sarcoidosis [4, 51, 52].

## 3. Histopathology and Immunology

The characteristic finding of sarcoidosis is the presence of noncaseating granulomas. The granuloma is comprised of mainly lymphocytes, epitheloid cells, macrophages, and fibroblasts in varying proportions. Some of the macrophages undergo differentiation to form epitheloid cells, mature, lose some of its phagocytic activity, and fuse to form multinucleated giant cells. During the early, acute phase, the core of the granuloma consists of mononuclear phagocytes in different stages of maturation and differentiation. This is surrounded by mainly CD4+ T cells and a few CD8+ T cells and B cells. CD8+ cells and fibroblasts become increased in number during the latter stages, as the granulomas become less active. Granulomas can also be identified in a number of other infectious and noninfectious states. Granulomas found in sarcoidosis are classically nonnecrotizing, tense and usually track along the lymphatics. Granulomas found in hypersensitivity pneumonitis or extrinsic allergic alveolitis are generally nonnecrotizing too, but are loosely formed and track along the airways. Granulomas seen in tuberculosis and histoplasmosis, however, are classically necrotizing tense and are distributed in no particular pattern. The lesions in berylliosis are very similar to those found in sarcoidosis, and therefore effort must be made to inquire about occupational exposure to beryllium and its various compounds [45].

Macrophages and dendritic cells present the antigens through the framework of the major histocompatibility complex (MHC) class II alleles to the T cells. They also secrete cytokines interleukin-12 (IL-12) which stimulates the maturation of CD4+ T lymphocytes to Th1 cells and interleukin-15 (IL-15) which promotes T lymphocyte propagation. CD4+ T lymphocytes which are the one of the primary cells involved in immunopathogenesis of sarcoidosis releases interleukin-2 (IL-2) and interferon-*γ* (IFN-*γ*). This further exaggerates the immune response and induces the proliferation and chemotaxis of T lymphocytes. Blood monocytes that arrive then undergo differentiation into exudates macrophages which in turn releases factors such as tumor necrosis factor-*α* (TNF-*α*) and interleukin-1*β* (IL-1*β*), which enhances the formation of a granuloma. These cells then further undergo organization and ultimately form noncaseating granulomas. It has been postulated that increased levels of transforming growth factor-*β* (TGF-*β*), insulin-like growth factor-1, and various changes in cytokine production (IL-4, IL-5) that now favor a Th2 cell pattern are responsible for progression to a fibrotic change in the granuloma [4, 53, 54]. 

## 4. Discussion

### 4.1. Review of Cases

A systematic review of the literature using the keywords “gastrointestinal sarcoidosis,” “esophageal sarcoidosis,” and “GI sarcoidosis” was done on Medline/Pubmed, Google Scholar, and the directory of Open access journals and by reviewing references of earlier published case reports. Our search yielded twenty-three documented cases of esophageal involvement in sarcoidosis. A summary of these reported cases can be found in Table 1. Thirteen (56.5%) of the published cases were recognized in women. The patient's ages varied from 29 years to 82 years with a median age at onset of symptoms being 48 years. The average age among men was 50 years and among women was 46 years.

Dysphagia has been reported to be the most common symptom in patients with esophageal sarcoidosis [36]. This closely correlates with our observation, where twenty-one of the twenty-three patients (91.3%) presented with some degree of difficulty swallowing and features suggestive of achalasia. Other clinical manifestations among patients include weight loss, abdominal pain, odynophagia, hoarseness of voice/dysphonia and anemia (Table 2). Rustagi and Majumder [41] also reported the case of a 45-year-old male who presented with complaints of difficulty swallowing and severe chest pain for 2 days. Further investigation discovered a complete perforation of the esophagus. Additionally, among the reported cases, only one case, reported by Panosetti and Lehmann [27], suggests possible isolated involvement of the esophagus in sarcoidosis. Siegel et al. [21] reported an interesting case of a patient with extensive granulomatous disease as evidenced on a biopsy of the gastrocnemius muscle, with the atypical feature that her only symptom at presentation was dysphagia.

The esophageal involvement in sarcoidosis can be classified based upon two criteria: the level of involvement and the layer of involvement [34]. The lower esophagus was the most commonly involved (56.5%), as was seen in thirteen of the reported cases (Table 1). The upper esophagus was documented to be involved in 6 cases (26.1%). Pathological involvement of the esophagus in sarcoidosis has been divided into four types: superficial mucosal involvement, involvement of the esophageal musculature, direct myenteric involvement, and extrinsic compression [55].

- Superficial involvement of the mucosa may manifest macroscopically as mucosal hyperemia, discrete plaque-like or nodular lesions. Murdock and Jacob [35] presented the case of woman who was referred for evaluation of anemia. Upper endoscopy showed the macroscopic appearance of Barrett's esophagitis, the histological examination of which was consistent with the diagnosis of esophageal sarcoidosis. Samarasena et al. [40] reported the case of a man who presented with complaints of dysphagia. Upper endoscopy showed a mid esophageal stricture that was nearly circumferential in nature. Endoscopic ultrasound examination (EUS) confirmed the esophageal lesion as due to thickened mucosal and submucosal layers with the presence of enlarged lymph nodes.
- Myopathic involvement in esophageal sarcoidosis occurs due to infiltration of the skeletal muscles of the esophagus and posterior pharynx. Siegel et al. [21] reported the case of a woman who presented with symptoms of progressive dysphagia. Barium swallow showed significant narrowing at the level of pharyngoesophageal junction. Pressure recordings measured the resting cricopharyngeal pressure to be elevated. A surgical myotomy was performed which provided symptomatic relief. Histological examination of the cricopharyngeus muscle contained numerous noncaseating granulomas. Similarly, Nishikubo et al. [39] describe the case of a woman who underwent cricopharyngeal myotomy due to dysphagia. The muscle on pathological examination was found to be fibrotic and hypertrophic with microscopic evidence of noncaseating granulomas. It has been reported that 50–80% of patients with sarcoidosis have some degree of muscle involvement. However, symptomatic muscle involvement is manifested in only 0.5–2.3% [56]. Pathophysiologically, muscle involvement is classified into three distinct classes: nodular lesions, acute myositis, and chronic myopathy. Chronic myopathic type of muscle involvement has been reported to be the most common, with the disease most prominent in elderly women and characterized by progressive symmetrical weakness of the affected muscles [57]. A diffusely increased uptake pattern is noted on gallium scintigraphy, and muscle biopsy reveals muscle fibers with varying degree of fibrosis and interspersed microscopic granulomas [58]. The previously described case was consistent with features of the chronic myopathic type. Muscle involvement with nodular lesions is the least common type and most frequently affects the lower limbs. Nodules are painless and are usually not associated with muscle weakness [59]. Features of acute myositis include painful muscle swelling, hypertrophy, and varying degrees of contractures, and this is characteristically seen in younger patients and is associated with erythema nodosum [57].
- Direct involvement of the enteric nervous plexus can cause dysphagia, and the clinical picture can mimic that of achalasia. Dufresne et al. [28] described a patient who presented with complaints of a sensation of food sticking in her lower esophagus. Motility studies that were done due to nonresolution of symptoms showed absence of peristaltic waves of the lower esophagus. The patient underwent a modified Heller operation and microscopic examination of the esophagus revealed a diffuse inflammatory infiltration of the myenteric nerve plexus, resulting in almost complete demyelinization, loss of axons, and active degeneration. Wasfi et al. [36] reported the rare case of a patient who presented with worsening dysphagia as a result of both extrinsic compression by enlarged mediastinal nodes and due to neuromuscular dysfunction due to direct infiltration. Previous studies have reported that the nervous system is involved in approximately 5% of cases, and in postmortem examination, the rates are as high as 15–27% [39]. Sarcoidosis is also known to affect the cranial nerves, with the facial and optic nerves most commonly involved, followed by the collective involvement of cranial nerves IX, X, XI [60]. Hardy et al. [23] described a patient who presented with dysphagia, dysphonia, weight loss, and difficulty clearing her throat accompanied by examination findings of ptosis and sluggish ocular movements. Histopathologic examination of the esophagus showed evidence of noncaseating granulomas; however, her other symptoms were thought to be due to the involvement of cranial nerves III, X, and XII.
- Dysphagia as a consequence of extrinsic compression from enlarged mediastinal lymph nodes due to sarcoidosis has also been described. Cook et al. [24] reported the case of a patient who presented with dysphagia. Further evaluation showed the extrinsic compression of the esophagus by enlarged mediastinal lymph nodes which on biopsy showed evidence of noncaseating granulomas. The patient was treated with steroids which resulted in resolution of her symptoms and a repeat esophagram showing no evidence of extrinsic compression. The patient described by Rustagi and Majumder [41] presented with an esophageal perforation which occurred as a result of an extremely rare complication of mediastinal lymph node hypertrophy causing esophageal erosion, necrosis, and perforation.

Diagnosis of sarcoidosis hinges upon establishing a compatible clinical and histopathologic picture whilst ruling out other granulomatous diseases. A detailed history including particulars about environmental and occupational exposure is key to identifying other granulomatous diseases. A detailed physical exam aids in assessing the extent of involvement of other organ systems. A baseline slit lamp examination to diagnose subclinical uveitis and measurement of 24 hour urine calcium to detect subclinical hypercalciura, are also recommended in all patients. Cardiac involvement, though rare, can cause life-threatening arrhythmias and therefore electrocardiograms are recommended at baseline and at frequent intervals [4]. A complete blood count, liver-related tests, and renal function tests are inexpensive initial tests that are frequently suggested. Since the lungs are the most frequently involved organ, a chest radiograph is advisable in all patients. Serum ACE levels and the Kveim-Siltzbach skin test are infrequently performed, but they lack both sensitivity and specificity. The cornerstone to diagnose esophageal involvement in sarcoidosis secondary to direct granulomatous infiltration is by performing an upper endoscopy and providing adequate tissue for histopathologic examination. Identification of granulomas should lead the physician to rule out other diseases such as Crohn's disease, tuberculosis, histoplasmosis, adenocarcinoma, foreign body causing a foreign body reaction, and tertiary syphilis. A review of 42 patients who were diagnosed with granulomas on gastric biopsy by Shapiro et al. [61] found the most common diagnosis to be Crohn's disease in 55% followed by sarcoidosis in 21% [62]. A barium swallow study can provide valuable information regarding the status of the esophageal sphincters and peristalsis. Computed tomography using contrast may be useful if an upper endoscopy is inconclusive and can help visualize enlarged lymph nodes. The oropharyngeal phase of swallowing can be adequately defined with a videofluoroscopic swallowing study. Esophageal manometry can provide invaluable information regarding contractile features of the upper gastrointestinal tract. Intraluminal impedence measurement is an alternative to fluoroscopy and can evaluate bolus transit through the esophagus [63]. Endoscopic ultrasound (EUS) can help to characterize and obtain samples of enlarged lymph nodes for histopathologic analysis in cases of extrinsic compression of the esophagus. Newer studies have demonstrated increased tracer uptake on fludeoxyglucose PET scans, corresponding with areas of known sarcoidosis involvement especially the heart, abdomen, and brain; however, similar readings can be demonstrated in other malignancies such as lymphomas [64]. Tracers such as fluoro-alpha-methyltyrosine have shown promise in making this distinction; however, more research is needed [3].

### 4.2. Treatment

There have been no clinical trials comparing the efficacy of the different treatment modalities for the treatment of esophageal sarcoidosis given the rarity of the disease. Dietary modifications, proton pump inhibitors, and surgical and corticosteroid therapy have been used to treat esophageal sarcoidosis. 

#### 4.2.1. Corticosteroids

The role of corticosteroids in the treatment of gastrointestinal sarcoidosis is not clear. However, considering the presence of systemic involvement in almost all cases of esophageal sarcoidosis and the observation that esophageal inflammation secondary to granulomatous inflammation seems to respond to corticosteroids, oral steroids remain the first-line therapy [40]. It has been reported that in the case of systemic gastric sarcoidosis, corticosteroid therapy leads to a favorable therapeutic response in 66% of the patients [55, 65].

Besides inhibiting the production of inflammatory cytokines such as IL-1, IL-2, IFN-*α*, and TNF, it also inhibits the formation of proinflammatory enzymes including collagenase and plasminogen activator. Moreover, corticosteroids have a negative impact on lymphocyte proliferation and delayed hypersensitivity reactions. The goal of steroid therapy in systemic sarcoidosis is to relieve symptoms thus improving quality of life and to halt the progression of disease and irreversible organ damage [4]. There have been no consensus guidelines regarding initial dosing and duration of therapy. However, currently various authors recommend starting therapy at 20 mg to 40 mg of oral prednisone daily [16, 55, 66]. After initiation of steroid therapy, patients should be followed up closely, preferably within 8 to 12 weeks to determine response and assess for side effects. Based on clinical response, a slow taper of the corticosteroid dose is initiated to a maintenance dose of 10 mg to 15 mg over a period of six months, while ensuring that symptoms do not relapse [66]. Patients are then reevaluated over time and an attempt should be made to withdraw oral steroid therapy completely. Unfortunately some patients require treatment to be restarted due to relapse of symptoms. 

#### 4.2.2. Surgery

The aim of surgical treatment is to provide symptomatic relief, eradicate outflow tract obstruction, and possibly prevent acid reflux. Cricopharyngeal myotomy can be used for the treatment of both oropharyngeal dysphagia and hypopharyngeal (Zenker's) diverticulum. Cricopharyngeal myotomy was first performed by Kaplan et al. in 1951 for the treatment of post-poliomyelitis dysphagia and it has since then been increasingly used for dysphagia due to various causes [67]. The procedure decreases the tone of the resting sphincter and facilitates flow. Many studies have shown that the response rates are over 60%; however, most of them lack authentic validation [63]. Moreover, the benefits of performing a cricopharyngeal myotomy are less certain in cases of oropharyngeal dysphagia due to neurogenic causes as opposed to those due to obstruction.

Esophageal myotomy to relieve achalasia was first reported by Ernest Heller in 1913 wherein both the anterior and posterior esophageal sphincters were cut. A modification of this technique involves only the dissection of the anterior muscle fibers and this has become the standard surgical procedure [68]. However, in recent times, laparoscopic Heller's myotomy coupled with a partial fundoplication to combat secondary reflux has become the preferred surgical procedure. Patti et al. [69] found that the laparoscopic approach was associated with postoperative reflux in 10% patients as compared to 60% undergoing thoracoscopic approach, while still having superior dysphagia relief rates (77% versus 70% for thoracoscopic patients). Similar rates were also obtained by Stewart et al. [70], who also showed that the laparoscopic approach was associated with shorter operative duration and speedier recovery.

Botulinum toxin is an irreversible inhibitor of acetylcholine release from presynaptic cholinergic terminals and this helps lower the lower esophageal sphincter tone. However, since new axon growth and regeneration counteracts this effect over time, treatment needs to be repeated at regular intervals. Multiple injections into the lower esophageal sphincter as a result of repeat treatment can cause local inflammation and fibrosis, further limiting the usefulness of this therapy. This option is thus reserved for older patients or those who are not ideal candidates for either surgical or corticosteroid therapy.

#### 4.2.3. Immunosuppressive Agents

Due to the rarity of esophageal involvement in sarcoidosis, the role of immunosuppressive therapy has not been defined. However, their use must be reserved for those patients who experience side effects due to systemic steroid therapy or in those with worsening of symptoms despite corticosteroids. Methotrexate is the most commonly used immunosuppressive agent in systemic sarcoidosis. Methotrexate is believed to decrease the antigen stimulated proliferation of lymphocytes and exerts its anti-inflammatory through adenosine accumulation [4, 71]. The beneficial effect of methotrexate in sarcoidosis manifests slowly and may take as long as six months. Patients with musculoskeletal and cutaneous involvement seem to report the greatest benefit. Other cytotoxic drugs infrequently used in the treatment of systemic sarcoidosis include azathioprine, cyclophosphamide, mycophenolate and leflunomide. Further research is, however, needed to validate its efficacy in esophageal sarcoidosis. 

#### 4.2.4. Antitumor Necrosis Factor *α* Therapy

As mentioned earlier, TNF production is a fundamental part of the immunologic process of sarcoidosis. A recent study found TNF to be secreted at higher levels in a subset of patients with refractory sarcoidosis. These observations have led to the logical idea that anti-TNF-*α* therapies may be valuable agents in the treatment of sarcoidosis [72]. Thalidomide is an agent which suppresses the release of TNF. Currently, it has only been used in treatment of chronic cutaneous sarcoidosis, as its use for treatment of other organ system involvement would need higher dosing regimens which would be limited by drug toxicity. Infliximab (chimeric monoclonal antibody against TNF-*α*), etanercept (soluble TNF receptor that binds to TNF in circulation), and adalimumab (humanized monoclonal antibody against TNF) are agents that are rarely used in the treatment of sarcoidosis. Few clinical series and trials have shown benefit of infliximab in the treatment of refractory sarcoidosis, including extrapulmonary manifestations [73]. Given that esophageal involvement in sarcoidosis is almost always accompanied by manifestations in various other organ systems, these agents may prove to be of significant benefit in the future. Any patient being considered for treatment with infliximab must be screened for untreated or latent Tuberculosis (TB) as reactivation of latent TB with use of anti-TNF-*α* agents has been described in the literature [74]. 

## 5. Conclusion

Gastrointestinal sarcoidosis is an extremely infrequent disease with clinically significant involvement seen in less than 1% of patients with sarcoidosis. The diagnosis of esophageal sarcoidosis can be challenging, not only because of the rarity of the disease but also because of the need for histopathologic evidence and the necessity to exclude other granulomatous diseases. It is likely that esophageal sarcoidosis is under-diagnosed due to the slow progression of the disease and difficulties in diagnosing the same. Upper endoscopy, esophageal manometry and endoscopic ultrasound are some valuable tools that can aid in timely diagnosis and therapeutic intervention. Corticosteroid therapy and various surgical techniques are most commonly used to relive symptoms of dysphagia secondary to esophageal involvement. The routine use of disease modifying agents including anti-TNF-*α* drugs in esophageal sarcoidosis will need further research and intense scrutiny. Clinicians must be cognizant of this rare but clinically important disease process and an interdisciplinary team approach is essential for a favorable outcome. 

## Figures and Tables

**Table 1:** Summary of case reports of esophageal sarcoidosis.

Cases	Sex of patient	Age at onset of symptoms	Involvement and cause	Symptoms	Treatment	Presence of systemic involvement
Kerley [20]	F	Not known	Direct infiltration of lower esophagus	Dysphagia	Not known	Yes
Siegel et al. [21]	F	59	Direct infiltration of pharyngoesophageal junction	Dysphagia and weight loss	Surgical myotomy	Yes
Polachek and Marte [22]	M	65	Direct infiltration of lower esophagus	Weight loss and abdominal pain	Steroid therapy	Yes
Hardy et al. [23]	F	31	Direct infiltration of lower esophagus	Dysphagia, weight loss, and dysphonia	Steroid therapy	Yes
Cook et al. [24]	F	29	Extrinsic compression of mid esophagus	Dysphagia	Steroid therapy	Yes
Wiesner et al. [25]	F	33	Direct infiltration of lower esophagus	Dysphagia	Surgical resection	Yes
Davies [26]	M	49	Direct infiltration of upper esophagus	Dysphagia	Not known	Yes
Panosetti and Lehmann [27]	F	71	Direct infiltration of pharyngoesophageal junction	Dysphagia	Surgical myotomy	No
Dufresne et al. [28]	F	46	Neural invasion of the lower esophagus	Dysphagia	Surgical myotomy	Yes
Aronson et al. [29]	M	82	Neural invasion of lower esophagus	Dysphagia and hoarseness	Steroid therapy	Yes
Nidiry et al. [30]	F	44	Neural invasion of lower esophagus	Dysphagia	Steroid therapy	Yes
Cappell [31]	M	42	Extrinsic compression of middle esophagus	Dysphagia	Not known	Yes
Boruchowicz et al. [32]	F	30	Neural invasion of lower esophagus	Dysphagia	Surgical myotomy	Yes
Geissinger et al. [33]	F	40	Possible neural involvement of lower esophagus	Dysphagia and weight loss	Steroids and proton pump inhibitors	Yes
Lukens et al. [34]	F	48	Direct infiltration of lower esophagus	Dysphagia	Botulinum toxin injection possibly followed by steroid therapy	Yes
Murdock and Jacob [35]	F	54	Direct infiltration of lower esophagus	Anemia and Barrett's esophagus	Proton pump inhibitor therapy	Yes
Wasfi and Margolis [36]	M	45	Neural invasion and extrinsic compression of upper esophagus	Dysphagia	Steroid therapy	Yes
Ohshimo et al. [37]	M	64	Direct infiltration of pharyngoesophageal junction	Dysphagia	Surgical myotomy	Yes
Bredenoord et al. [38]	M	29	Neural invasion of the lower esophagus	Dysphagia and weight loss	Steroid therapy	Yes
Nishikubo et al. [39]	F	73	Direct infiltration of cricopharyngeal muscle	Dysphagia	Surgical myotomy	Yes
Samarasena et al. [40]	M	46	Direct infiltration of mid esophagus causing stricture	Dysphagia	Not known	Yes
Rustagi and Majumder [41]	M	45	Extrinsic compression of mid esophagus	Dysphagia, chest pain, and esophageal rupture	Ivor Lewis's esophagectomy	Yes
Ruiz et al. [42]	M	37	Direct infiltration and neural invasion of lower esophagus	Dysphagia and odynophagia	Laparoscopic Heller's myotomy with fundoplication	Yes

**Table 2:** Most frequently documented symptoms at presentation.

Dysphagia	91.3%
Odynophagia	4.3%
Weight loss	21.7%
Anemia	4.3%
Abdominal and/or chest pain	8.7%
Hoarseness of voice	8.7%
